# Memory is preserved in older adults taking AT1 receptor blockers

**DOI:** 10.1186/s13195-017-0255-9

**Published:** 2017-04-26

**Authors:** Jean K. Ho, Daniel A. Nation, for the Alzheimer’s Disease Neuroimaging Initiative

**Affiliations:** Department of Psychology, University of Southern California, 3620 South McClintock Avenue, SGM 1010, Los Angeles, CA 90089-1061 USA

**Keywords:** Blood pressure, Antihypertensive medication, Angiotensin receptor blockers, Memory, Alzheimer’s disease

## Abstract

**Background:**

Prior work suggests that some but not all antihypertensive treatments may benefit cognition and risk for Alzheimer’s disease, independent of stroke. Angiotensin II receptor blockers (ARBs) have been highlighted as one antihypertensive drug class that may confer greatest benefit.

**Methods:**

The participants comprised 1626 nondemented adults, aged 55–91 years, recruited from Alzheimer’s Disease Neuroimaging Initiative sites. Three groups were compared: ARB users (HTN-ARBs), other antihypertensive drug users (HTN-Other), and normotensives. In post hoc analyses, we also examined (1) users of ARBs and angiotensin-converting enzyme inhibitors (ACEIs), (2) users of blood-brain barrier (BBB)-crossing ARBs and users of non-BBB-crossing ARBs, and (3) users of BBB-crossing ARBs and ACEIs (BBB crossers) and users of non-BBB-crossing ARBs and ACEIs (BBB noncrossers). Groups were compared regarding cognition and magnetic resonance imaging measures of brain volume and white matter hyperintensities (WMH), using analysis of covariance and multilevel models.

**Results:**

At baseline, the HTN-Other group performed worse than normotensives on Rey Auditory Verbal Learning Test (RAVLT) Immediate Recall (*p* = 0.002), Delayed Recall (*p* < 0.001), Recognition Memory (*p* = 0.001), and Trails A (*p* < 0.001) and B (*p* = 0.01). ARB users performed better than the HTN-Other group on Recognition Memory (*p* = 0.04) and worse than normotensives on Trails A (*p* = 0.04). The HTN-Other group performed worse than normotensives on Logical Memory Immediate (*p* = 0.02) and Delayed Recall over the 3-year follow-up (*p* = 0.007). Over the follow-up period, those taking BBB-crossing ARBs performed better than the HTN-Other group on AVLT Delayed Recall (*p* = 0.04), Logical Memory Immediate (*p* = 0.02), and Delayed Recall (*p* = 0.05). They also had fewer WMH than the HTN-Other group (*p* = 0.008) and those taking non-BBB-crossing ARBs (*p* = 0.05). There were no group differences in brain volume. Users of BBB-crossing medications (ARBs or ACEIs) showed better performance on AVLT Delayed Recall over time than all other groups, including normotensives (all *p* < 0.01), and had less WMH volume over time than the BBB noncrossers group (*p* = 0.03), although they had more WMH volume than normotensives (*p* = 0.01). The BBB noncrossers group performed worse than normotensives on Logical Memory Delayed Recall over time (*p* = 0.01), but the BBB crossers group was not significantly different (*p* = 0.13).

**Conclusions:**

Hypertensive participants demonstrated worse baseline memory and executive function, as well as greater memory decline, over the 3-year follow-up than normotensives, unless they were ARB users, who showed preserved memory compared with those taking other antihypertensive drugs. Users of BBB-crossing ARBs showed superior memory performance over time compared with other antihypertensive drug users and had less WMH volume. Users of BBB-crossing medications (ARBs or ACEIs) showed better list-learning memory performance over time than all other groups, including normotensives, and had less WMH volume over time than users of non-BBB-crossing medications. These findings demonstrate that ARBs, especially those of the BBB-crossing variety, are associated with greater memory preservation and less WMH volume than other antihypertensive medications.

**Electronic supplementary material:**

The online version of this article (doi:10.1186/s13195-017-0255-9) contains supplementary material, which is available to authorized users.

## Background

Hypertension is the most common condition observed in primary care and among the most treatable risk factors for illness [1]. Hypertension is also an established risk factor for cognitive decline, Alzheimer’s disease, and vascular dementia [2]. These and other findings support the vascular hypothesis of Alzheimer’s disease, which postulates that cerebrovascular disease and dysfunction, due to hypertension and other vascular risk factors, contribute to this dementing illness through effects on cerebral perfusion and blood-brain barrier (BBB) compromise [3, 4]. Therefore, work surrounding antihypertensive treatments and their possible salutary effects on preserving cognition through improved vascular health forms a promising area of research [5].

Hypertension and vascular risk factors have been linked to decreased cognitive function [6] beyond expected declines in processing speed and memory associated with normal aging [7]. For example, hypertension and diabetes mellitus have been found to be positively associated with cognitive decline in middle-aged adults, a decline that is greater than that experienced by healthier counterparts without these risk factors [8]. Midlife hypertension and elevations in serum cholesterol levels have also been linked to the development of mild cognitive impairment (MCI) in later life [9]. Relative to normotensive individuals, hypertensive persons exhibit worse performance in many domains of cognitive function, including episodic memory [10], working memory and executive function [11], attention and psychomotor speed [12], and language [13]. These cognitive deficits may be underpinned by hypertension-related vascular brain injury caused by alterations in cerebral hemodynamics and cerebral small vessel disease [14]. Functional implications of these vascular changes have been demonstrated in mouse models, where arterial hypertension has been found to increase the permeability and dysfunction of the BBB as well as to reduce cerebral blood flow [15]. Increased blood pressure, particularly pulse pressure, has also been linked to amyloid-β (Aβ) deposition [16, 17], tau-mediated neurodegeneration [18], and Alzheimer’s disease [19].

Despite these clear links between blood pressure and both dementia and Alzheimer’s disease, studies investigating cognitive benefits of blood pressure control have been mixed. Whereas some randomized controlled trials (RCTs)—Medical Research Council Elderly (MRC-Elderly), Systolic Hypertension in the Elderly Program (SHEP), Perindopril Protection against Recurrent Stroke Study (PROGRESS), Systolic Hypertension in Europe (SYST-EUR) trial, and the observational SYST-EUR 2 study—indicated some benefit, the Hypertension in the Very Elderly Trial assessing cognitive decline and dementia incidence (HYVET-COG), the Telmisartan Randomised AssessmeNt Study in ACE iNtolerant subjects with cardiovascular Disease (TRANSCEND), and the Ongoing Telmisartan Alone and in Combination with Ramipril Global Endpoint Trial (ONTARGET) as well as a Cochrane review did not [20, 21]. Of the former, it must be noted that the MRC-Elderly study [22] was not a double-blind trial and included only two longitudinal cognitive outcome measures. The PROGRESS study did not find a clear effect of hypertensive treatment on dementia, but the researchers did report that treatment reduced the risk of cognitive decline by approximately 20% and reduced the risk of “cognitive decline with recurrent stroke” by approximately 50% [23]. The study authors suggested that benefits of hypertensive treatment in their sample may have been the result of stroke prevention as opposed to direct influence on dementia or cognitive impairment [23]. In the SHEP study [24], a double-blind RCT, researchers found slight positive effects of treatment on cognitive, physical, and leisure functioning but failed to find any significant difference in the incidence of cognitive deterioration between participants on active hypertension treatment and placebo. However, a report on the reanalysis of these data indicated that differential dropout from the treatment and placebo groups may have biased these results toward a null effect [25].

In studies suggesting possible protective effects, individuals treated for hypertension have been found to have less Alzheimer’s disease neuropathology than both untreated hypertensive individuals and normotensive persons, evidenced in lower neuritic plaque counts and fewer neurofibrillary tangles of hyperphosphorylated tau protein [26]. Compared with individuals who have never taken any antihypertensive drugs, individuals treated with these medications exhibit decreased risk of all-cause dementia, with an 8% risk reduction for every year of use in individuals <75 years old, with similar estimates for Alzheimer’s disease [27]. Authors of a recent meta-analysis of 19 randomized trials and 11 studies examining the relationships among antihypertensive drug use, cognition, and dementia incidence provided support for the perspective that antihypertensive treatment may have beneficial effects on cognition [28]. Of the many antihypertensive drug classes available, angiotensin II receptor blockers (ARBs) were highlighted as the one class that may confer the greatest benefit [28].

Therefore, in the present study, we sought to investigate whether use of ARBs would confer protective effects on cognition greater than those of other antihypertensive drugs. Our specific hypotheses were that (1) normotensive individuals would show the best preservation of neuropsychological function, followed by ARB users, who in turn would outperform users of other antihypertensive drugs; (2) ARB users would show better memory and executive function than users of other antihypertensive drugs, in line with positive effects reported elsewhere [29, 30]; and (3) in comparison with users of other antihypertensive drugs, ARB users would show attenuation of cognitive decline over time.

## Methods

Data were obtained from the Alzheimer’s Disease Neuroimaging Initiative (ADNI) database (adni.loni.usc.edu). The primary goal of ADNI is to test whether neuroimaging, other biological markers, and clinical and neuropsychological assessments can be combined to measure the progression of MCI and early Alzheimer’s disease. ADNI is the result of efforts of many coinvestigators from a range of academic institutions and private corporations, and subjects have been recruited from more than 50 sites across the United States and Canada. Participants are recruited via newsletters, Internet-based communications, direct mail, and press releases. Inclusion criteria include aged 55–91 years, permitted medications stable for 4 weeks, study partner who can accompany participant to visits, Geriatric Depression Scale score less than 6, Hachinski Ischemic Scale score less than or equal to 4, adequate visual and auditory acuity, good general health, 6 grades of education or work history equivalent, and ability to speak English or Spanish fluently. Exclusion criteria for cognitively normal subjects and participants with MCI include any significant neurological disease or history of significant head trauma. For more information, *see* www.adni-info.org.

### Participants

The participants comprised 1626 nondemented ADNI-1, ADNI-Grand Opportunity, and ADNI-2 participants who were classified as either cognitively normal or having MCI at screening evaluation. Criteria for MCI were (1) subjective memory complaint reported by the participant or informant, (2) Mini Mental State Examination (MMSE) scores between 24 and 30 (inclusive), (3) global Clinical Dementia Rating of 0.5, (4) scoring below education-adjusted cutoffs for delayed free recall on Story A of the Wechsler Memory Scale–Revised (WMS-R) Logical Memory II subtest, and (5) general cognition and functional performance preserved to the extent that one would not qualify for a diagnosis of Alzheimer’s disease [31].

### Blood pressure medication groups

Eight hundred four participants (49.4% of the total sample) reported using at least one of eight classes of antihypertensive drugs: ARBs, angiotensin-converting enzyme inhibitors (ACEIs), β-blockers, calcium channel blockers, α_1_-adrenergic blockers, α_2_-agonists or other centrally acting drugs, diuretics, or direct vasodilators. Of these, 183 (11.2% of the total sample) were taking ARBs and were classified as ARB users (HTN-ARBs). Six hundred twenty-one (38.3% of the total sample) were taking other antihypertensive medications and were classified as users of other antihypertensive drugs (HTN-Other). The remaining 822 (50.5% of the total sample) had no documented history of hypertension and were classified as normotensive. To ensure that untreated hypertensive subjects were excluded, blood pressure measures were used to exclude those with values at or above stage 2 hypertension (160/100 mmHg; *n* = 40). We did not use the more conservative cutoffs recommended for treatment of older adults (150/90 mmHg), owing to the unreliability of a single blood pressure measure and the likelihood that the research environment may have artificially produced transient, mild elevations in blood pressure in normotensive individuals. Thus, the final sample consisted of 1586 participants (*see* Additional file 3).

Given that ACEIs also act on the renin-angiotensin-aldosterone system (RAAS), and given that the capability of medications to cross the BBB is related to drug efficacy, in post hoc analyses we also examined (1) users of ARBs and ACEIs (*n* = 464); (2) users of BBB-crossing ARBs of valsartan, telmisartan, and candesartan (*n* = 72 ARB crossers) and users of non-BBB-crossing ARBs of irbesartan, olmesartan, losartan, and eprosartan [32] (*n* = 102 ARB noncrossers); and (3) users of BBB-crossing ARBs and BBB-crossing ACEIs of captopril, fosinopril, lisinopril, perindopril, trandolapril, or zofenopril (*n* = 277 BBB crossers) and users of non-BBB-crossing ARBs and non-BBB-crossing ACEIs of benazepril, enalapril, moexepril, quinapril, and ramipril [33] (*n* = 187 BBB-noncrossers).

### Physiological, clinical, and genetic data

Physiological measures included seated brachial artery systolic and diastolic blood pressures. Pulse pressure was computed as systolic minus diastolic pressure. Data on blood pressure indices were available for 98.8% of the sample. Body mass index (BMI) was calculated as weight (in kilograms) divided by height (in meters) squared. Data on BMI were available for 98.8% of the sample. Blood samples were used to determine apolipoprotein E (APOE) ε4 allele carrier status. Participants were categorized as those with or without one or more copies of the APOE ε4 allele. APOE genotype data were available for 85.3% of the sample.

### Vascular risk factors

Vascular risk factor burden was assessed on the basis of medical history and physical examination at baseline and screening using criteria adapted from the Framingham Stroke Risk Profile [34] and Framingham Coronary Risk Profile [35]. Vascular risk factors included cardiovascular disease (myocardial infarction, intermittent claudication, angina, heart failure, or other evidence of coronary disease), dyslipidemia (low levels of high-density lipoprotein cholesterol, high levels of low-density lipoprotein cholesterol, or hypertriglyceridemia), type 2 diabetes, atrial fibrillation, evidence of carotid artery disease, and transient ischemic attack (TIA) or minor stroke.

### Neuropsychological battery

Cognitive measures included (1) WMS-R Logical Memory II subtest, immediate and delayed recall on Story A; (2) Rey Auditory Verbal Learning Test (RAVLT), total immediate recall score for trials 1–5, delayed recall score, and recognition score; (3) Wechsler Adult Intelligence Scale–Revised Digit Span forward and backward scores and spans; (4) Trail Making Tests A and B, times to completion; (5) Animal Fluency total score; (6) Vegetable Fluency total score; and (7) Boston Naming Test (BNT) total score (*see* Table 2).

### Brain volume estimation

Participants had magnetic resonance imaging (MRI) at 1.5 T. The data were obtained following a standardized MRI protocol (http://adni.loni.usc.edu/about/centers-cores/mri-core/) that was developed following an initiative to evaluate and compare three-dimensional T1-weighted sequences for morphometric analyses [36]. Each participant had two T1-weighted MRI scans collected using a sagittal volumetric magnetization prepared rapid gradient echo sequence with the following acquisition parameters: echo time of 4 milliseconds, repetition time of 9 milliseconds, flip angle of 8 degrees, acquisition matrix size of 256 × 256 × 166 (*x*-, *y*-, and *z*-dimensions). The normal voxel size was 0.94 × 0.94 × 1.2 mm.

Anatomical boundaries of the hippocampi and ventricles were traced using a semiautomated brain-mapping method developed on the basis of a high-dimensional fluid transformation algorithm [37]. White matter hyperintensities (WMH) were detected on coregistered T1-, T2-, and proton density-weighted images using automated methods described elsewhere [38, 39]. To correct for observed kurtosis in the distribution of the WMH volumes, we applied a log transformation prior to analyses.

### Statistical analyses

#### Cross-sectional analyses

Data were screened for departures from normality using indices of skewness and kurtosis. Scores from the Trail Making Test A and B and the BNT exhibited significant skewness, which was corrected by log transformation. Groups were compared on clinical and demographic variables using chi-square tests for nominal variables and one-way analysis of variance (ANOVA) for continuous variables. Performance on neuropsychological measures, as well as measures of hippocampal, ventricular, and WMH volume were compared using one-way ANOVA and analysis of covariance, controlling for age, sex, education, APOE ε4 allele carrier status, and BMI. Both analyses produced the same pattern of results; hence, only corrected analyses are presented in Table 2. Group differences were examined using post hoc least significant difference tests and chi-square analyses. All analyses were two-tailed, with significance set at *p* < 0.05.

#### Longitudinal analyses

To examine group differences and time × group interactions for cognitive performance and brain volume measures, multilevel model analyses were conducted with compound symmetric covariance structure and restricted maximum likelihood estimation. Time was entered as a random effect. Group, time × group, age, sex, education, APOE ε4 carrier status, and BMI were entered as fixed factors. Participants were clustered by site to account for local prescribing practice. Analyses were also conducted with the inclusion of systolic blood pressure and the number of antihypertensive medications taken at baseline as additional covariates. The broad pattern of results remained unchanged; hence, results without these two covariates are presented. Given that this was an exploratory study, with no prior studies having investigated the use of ARBs in relation to comprehensive measures of neuropsychological function, we did not apply multiple comparison corrections. Analyses were two-tailed with α set at *p <* 0.05. All analyses were performed with IBM SPSS for Mac OS X version 21.0 (IBM, Armonk, NY, USA) and SAS version 9.4 (SAS Institute Inc., Cary, NC, USA) software.

## Results

### Physiological, clinical, and genetic data

Three groups were compared: ARB users (HTN-ARBs), users of other antihypertensive drugs (HTN-Other), and a normotensive group that did not take any antihypertensive drugs (*see* “Blood pressure medication groups” section above and Additional files 1 and 2 for the specific medications used per group). As shown in Table 1, there were significant group differences in sex and diastolic blood pressure (*p* < 0.05), age, BMI, systolic blood pressure, pulse pressure (all *p* < 0.001), and education (*p* = 0.005). The normotensive group had significantly fewer males than the HTN-Other group (*p* = 0.004), was significantly younger (*p* < 0.05), and had a higher education level (*p* < 0.05). It also had better vascular health, as demonstrated by significantly lower diastolic blood pressure (*p* < 0.05), BMI, systolic blood pressure, and pulse pressure (all *p* < 0.001), than both treated hypertensive groups. At the 3-year follow-up, normotensive individuals continued to have significantly lower systolic blood pressure than both hypertensive groups (HTN-ARBs *p* = 0.01, HTN-Other *p* < 0.001) and lower pulse pressure than the HTN-Other group (*p* < 0.001).

**Table 1:** Clinical and demographic data

Characteristics	Total (*n* = 1586)	Normotensive (*n* = 782)	HTN-ARBs (*n* = 183)	HTN-Other (*n* = 621)	*F* or χ^2^ value	*p* Value
Baseline clinical/demographic characteristics						
Age, years	73.1 (7.2)	71.9 (7.3)	73.3 (6.8)	74.6 (6.8)	25.737	**<0.001**
Education, years	16.1 (2.8)	16.3 (2.7)	15.9 (3.2)	15.9 (2.8)	5.282	**0.005**
Male sex, %	53.8%	50.6%	51.6%	58.4%	8.685	**0.013**
APOE genotype, ε4 allele-positive, %	42.3%	43.4%	36.8%	42.5%	2.365	0.306
MCI diagnosis, %	60.3%	59.8%	54.4%	62.7%	4.216	0.121
BMI, kg/m^2^	27.1 (4.8)	26.4 (4.6)	28.0 (4.8)	27.6 (4.8)	14.844	**<0.001**
Systolic BP, mmHg	134.1 (15.7)	130.9 (14.4)	138.5 (16.4)	136.7 (16.2)	32.318	**<0.001**
Diastolic BP, mmHg	74.5 (9.4)	73.9 (8.9)	75.5 (9.7)	75.0 (9.8)	3.467	**0.031**
Pulse pressure, mmHg	59.6 (14.2)	57.1 (13.1)	63.0 (15.5)	61.7 (14.6)	25.014	**<0.001**
Baseline vascular risk factors						
Cardiovascular disease	11.2%	3.7%	17.6%	18.8%	87.645	**<0.001**
Dyslipidemia	46.2%	37.6%	59.3%	53.2%	48.249	**<0.001**
Type 2 diabetes	8.6%	3.7%	23.1%	10.5%	75.236	**<0.001**
Atrial fibrillation	3.3%	1.8%	3.3%	5.1%	12.293	**0.002**
Carotid artery disease	0.6%	0.4%	0.5%	1.0%	1.888	0.389
TIA/minor stroke	2.9%	1.3%	3.3%	4.8%	15.569	**<0.001**
3-Year follow-up blood pressure data	**Total** **(** ***n*** ** = 779)**	**Normotensive** **(** ***n*** ** = 380)**	**HTN-ARBs** **(** ***n*** ** = 89)**	**HTN-Other** **(** ***n*** ** = 310)**		
Systolic BP, mmHg	131.9 (16.9)	129.0 (15.8)	133.9 (16.2)	134.9 (17.7)	11.602	**<0.001**
Diastolic BP, mmHg	72.6 (9.6)	72.0 (9.3)	74.0 (10.2)	73.0 (9.8)	1.843	0.159
Pulse pressure, mmHg	59.3 (14.7)	56.9 (14.0)	59.9 (13.2)	61.9 (15.5)	10.203	**< 0.001**

*Abbreviations: APOE* Apolipoprotein E, *BMI* Body mass index, *BP* Blood pressure, *HTN-ARBs* Participants who took angiotensin II receptor blockers, *HTN-Other* Participants who took other antihypertensive drugs that were not angiotensin II receptor blockers, *MCI* Mild cognitive impairment, *TIA* Transient ischemic attack

Data are summarized as mean (SD), unless otherwise indicated

Significant differences (*p* < 0.05) among medication groups are indicated by boldface type

### Vascular risk factors

There were significant group differences in history of cardiovascular disease, dyslipidemia, type 2 diabetes, TIA/minor stroke (all *p* < 0.001), and atrial fibrillation (*p* = 0.002). Both hypertensive groups had significantly more participants with prior cardiovascular disease (*p* < 0.001), dyslipidemia (*p* < 0.001), and type 2 diabetes (*p* < 0.001) than the normotensive group. The HTN-Other group also had significantly more participants with a history of atrial fibrillation (*p* < 0.001) and TIA/stroke (*p* < 0.001) than the normotensive group. The ARB group had significantly more participants with type 2 diabetes than the HTN-Other group (*p* < 0.001).

### Cross-sectional analyses

#### Neuropsychological function

**Fig. 1:**
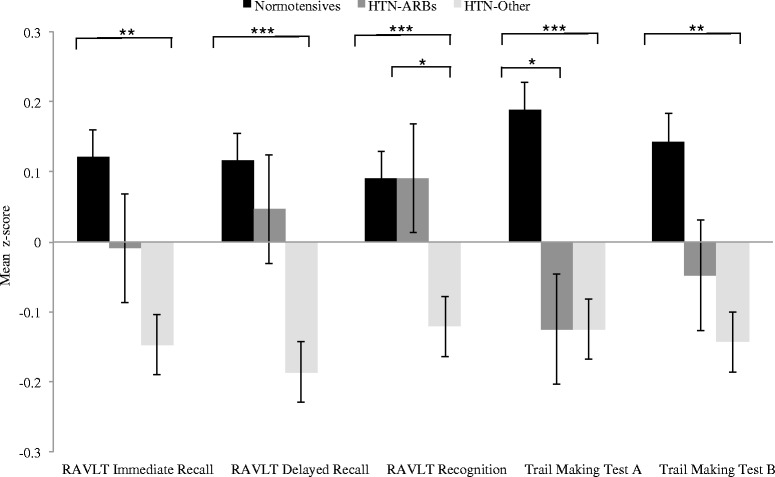
Baseline neuropsychological performance for the three medication groups across the total sample. Raw scores of mean performance were converted to z-scores using means and standard deviations of the final sample. Inverse z-scores for log-transformed Trail Making Test A and Trail Making Test B scores (time to completion) are presented such that positive values indicate better performance. The participants who took other antihypertensive drugs that were not angiotensin II receptor blockers (HTN-Other) performed worse on tests of memory, attention, and executive function than normotensive subjects. However, angiotensin II receptor blocker users (HTN-ARBs) did not differ from normotensive subjects on memory function and demonstrated better recognition memory than those taking other antihypertensive medications. * *p* < 0.05, ** *p* < 0.01, *** *p* < 0.001. *RAVLT* Rey Auditory Verbal Learning Test

As shown in Table 2, there were significant group differences on measures of memory, attention, and executive function after correcting for covariates. The HTN-Other group performed worse than the normotensive group on all of these measures (*see* Fig. 1): RAVLT Immediate Recall (*p* = 0.002), RAVLT Delayed Recall (*p* < 0.001), RAVLT Recognition (*p* = 0.001), Trail Making Test A (*p* < 0.001), and Trail Making Test B (*p* = 0.01). The HTN-ARBs group performed worse than the normotensive group only on Trail Making Test A (*p* = 0.04). The HTN-ARBs group performed significantly better than the HTN-Other group on a measure of memory (RAVLT Recognition *p* = 0.04) and displayed a nonsignificant trend toward outperforming the HTN-Other group on RAVLT Delayed Recall (*p* = 0.058).

**Table 2:** Baseline neuropsychological data

Neuropsychological test	Total (*n* = 1586)	Normotensive (*n* = 782)	HTN-ARBs (*n* = 183)	HTN-Other (*n* = 621)	*F*-Value	*p* Value
Memory						
LM Immediate Recall	10.6 (4.3)	10.7 (4.4)	10.9 (4.3)	10.4 (4.2)	0.269	0.764
LM Delayed Recall	8.5 (5.0)	8.6 (5.1)	9.2 (5.0)	8.3 (4.8)	1.987	0.137
RAVLT Immediate Recall	38.2 (11.6)	39.6 (11.8)	38.1 (11.8)	36.5 (11.1)	4.986	**0.007**
RAVLT Delayed Recall	5.3 (4.3)	5.8 (4.4)	5.5 (4.4)	4.5 (3.9)	9.642	**<0.001**
RAVLT Recognition	11.4 (3.3)	11.7 (3.2)	11.7 (3.1)	11.0 (3.5)	5.692	**0.003**
Attention/executive function						
Digit Span forward score	8.4 (2.0)	8.5 (1.9)	8.1 (2.2)	8.4 (2.1)	1.100	0.402
Digit Span forward span	6.6 (1.1)	6.7 (1.0)	6.5 (1.1)	6.6 (1.1)	0.953	0.386
Digit Span backward score	6.5 (2.1)	6.6 (2.2)	6.5 (2.1)	6.4 (2.1)	0.121	0.886
Digit Span backward span	4.8 (1.2)	4.8 (1.2)	4.7 (1.1)	4.7 (1.2)	0.021	0.979
Trail Making Test A score^a^	1.56 (0.16)	1.53 (0.15)	1.58 (0.16)	1.58 (0.16)	8.363	**<0.001**
Trail Making Test B score^a^	1.97 (0.21)	1.94 (0.21)	1.98 (0.20)	2.00 (0.21)	3.900	**0.020**
Language						
Animal Fluency	18.4 (5.5)	18.7 (5.4)	18.4 (5.3)	18.0 (5.7)	0.173	0.841
Vegetable Fluency	12.2 (4.1)	12.3 (3.9)	12.2 (4.2)	12.1 (4.3)	0.206	0.814
BNT^a^	0.48 (0.33)	0.47 (0.33)	0.47 (0.33)	0.51 (0.33)	1.385	0.251

*Abbreviations:* *BNT* Boston Naming Test, *HTN-ARBs* Participants who took angiotensin II receptor blockers, *HTN-Other* Participants who took other antihypertensive drugs that were not angiotensin II receptor blockers, *LM* Logical Memory, *RAVLT* Rey Auditory Verbal Learning Test

Data are summarized as mean (SD), unless otherwise indicated. All scores were corrected for age, sex, education level, BMI, and apolipoprotein ε4 allele carrier status. Significant differences (*p* < 0.05) among medication groups are indicated by boldface type

^a^Scores were log-transformed and are presented to two decimal places

#### Brain MRI measures

There were significant group differences in WMH volume [*F*(2, 1252) = 4.41, *p* = 0.01, *η*_p_^2^ = 0.01] from the HTN-Other group exhibiting significantly greater WMH volume than the normotensive group (*p* = 0.004). There were no differences in ventricular volume [*F*(2, 552) = 0.22, *p* = 0.81], left hippocampal volume [*F*(2, 552) = 0.48, *p* = 0.62], or right hippocampal volume [*F*(2, 552) = 0.93, *p* = 0.40].

### Longitudinal analyses

#### Neuropsychological function

**Fig. 2:**
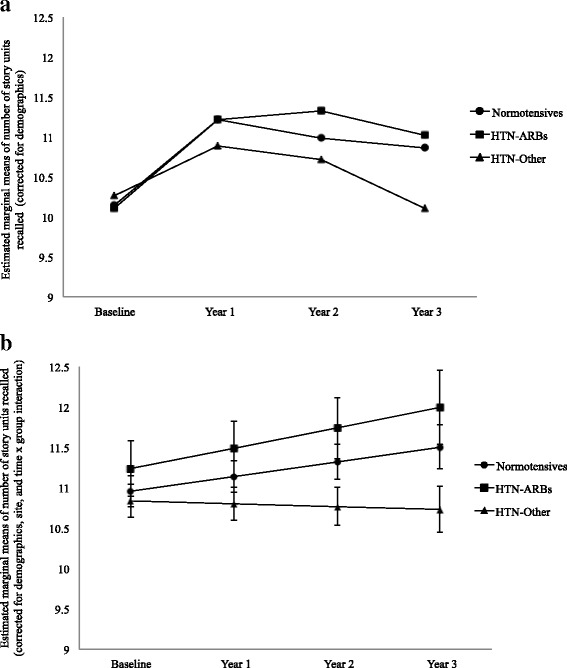
Logical Memory Immediate Recall performance over the 3-year follow-up period. **a** Estimated marginal means after correction for demographics only. **b** Means adjusted for demographics, Alzheimer’s Disease Neuroimaging Initiative site, and time × group interaction. After all adjustments, the participants who took other antihypertensive drugs that were not angiotensin II receptor blockers (HTN-Other) showed declining performance over time that was significantly worse than that of normotensive subjects and the participants who took angiotensin II receptor blockers (HTN-ARBs), with both of the latter groups showing improvement. The HTN-ARBs group was no different from normotensive subjects

There were significant time × group interactions for both measures of Logical Memory, Immediate Recall [*F*(2, 1772) = 3.63, *p* = 0.03], and Delayed Recall [*F*(2, 1767) = 3.72, *p* = 0.02]. As shown in Fig. 2, the HTN-Other group showed significantly worse performance on Immediate Recall over the 3-year follow-up than normotensive subjects [β = −0.22, *t*(1772) = −2.39, *p* = 0.02], as well as compared with the HTN-ARBs group [β = −0.29, *t*(1772) = −2.02, *p* = 0.04]. The HTN-ARBs group was no different from the normotensive group [β = 0.07, *t*(1772) = 0.48, *p* = 0.63].

**Fig. 3:**
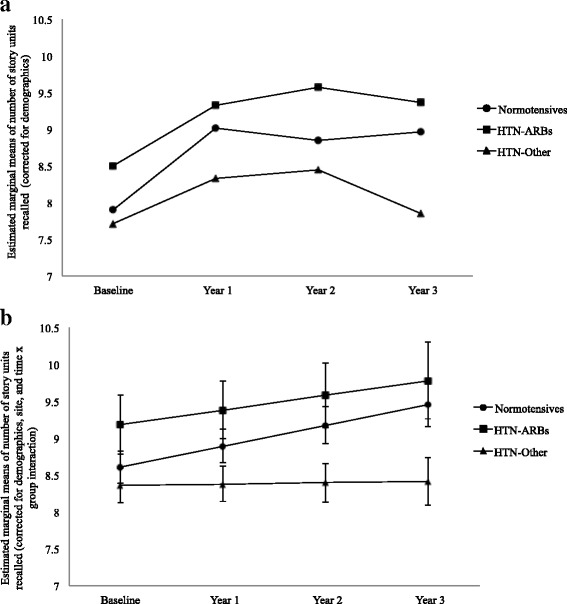
Logical Memory Delayed Recall performance over the 3-year follow-up period. **a** Estimated marginal means after correction for demographics only. **b** Means adjusted for demographics, Alzheimer’s Disease Neuroimaging Initiative site, and time × group interaction. After all adjustments, the participants who took other antihypertensive drugs that were not angiotensin II receptor blockers (HTN-Other) showed stable performance over time that was significantly worse than the performance of normotensive subjects, who showed improvement over time. The participants who took angiotensin II receptor blockers (HTN-ARBs) also improved over time and were no different from normotensive subjects

As shown on Fig. 3, for Logical Memory Delayed Recall, the HTN-Other group performed worse than normotensive subjects over the follow-up period [β = −0.27, *t*(1767) = −2.72, *p* = 0.007]. The HTN-ARBs group was no different from normotensive subjects [β = −0.08, *t*(1767) = −0.57, *p* = 0.57].

There were significant effects of group for RAVLT Immediate Recall [*F*(2, 3006) = 3.61, *p* = 0.03], RAVLT Delayed Recall [*F*(2, 2995) = 5.94, *p* = 0.003], RAVLT Recognition [*F*(2, 2991) = 3.23, *p* = 0.04], Trail Making Test A [*F*(2, 2998) = 7.63, *p* = 0.0005], and Trail Making Test B performance [*F*(2, 2932) = 4.51, *p* = 0.01]. The HTN-Other group performed significantly worse than the normotensive subjects on all these measures (RAVLT Immediate Recall *p* = 0.008, RAVLT Delayed Recall *p* = 0.0006, RAVLT Recognition *p* = 0.03, Trail Making Test A *p* = 0.0007, Trail Making Test B *p* = 0.006). The HTN-ARBs group performed worse than normotensive subjects on Trail Making Test A (*p* = 0.004) and Trail Making Test B (*p* = 0.04) only. The results were no different from those of normotensive subjects on all RAVLT measures.

#### Brain MRI measures

There were significant main effects of time for WMH volume [*F*(1, 2277) = 32.11, *p* < 0.0001], left hippocampal volume [*F*(1, 1059) = 1245.07, *p* < 0.0001], right hippocampal volume [*F*(1, 1059) = 1664.23, *p* < 0.0001], and ventricular volume [*F*(1, 1059) = 1045.76, *p* < 0.0001]. Ventricular volume and WMH volume significantly increased over time, whereas both left and right hippocampal volume significantly decreased over time (all *p* < 0.0001). There were no significant interactions between time and group and no significant main effects of group. However, there was a nonsignificant group trend for WMH volume [*F*(2, 2279) = 2.61, *p* = 0.07]. The HTN-Other group showed a nonsignificant trend toward having greater WMH volume than the HTN-ARBs group (*p* = 0.08) and normotensive subjects (*p* = 0.05).

### Post hoc exploratory analyses

#### Drugs affecting the renin-angiotensin-aldosterone-system

In post hoc multilevel model analyses, we examined whether users of RAAS drugs (ACEIs and ARBs) performed as well as the HTN-ARBs group. When this group (the ARB-ACEI group) was compared with users of other antihypertensive drugs (HTN-Other) and normotensive subjects, a significant time × group interaction was found for Logical Memory Delayed Recall [*F*(2, 1767) = 3.00, *p* = 0.05]. The ARB-ACEI group performed worse than normotensive subjects on this measure over time [β = −0.23, *t*(1767) = −2.17, *p* = 0.03]. There was a trending effect for the HTN-Other group performing worse than normotensive subjects [β = −0.22, *t*(1767) = −1.81, *p* = 0.07].

There were significant effects of group for RAVLT Immediate Recall [*F*(2, 3006) = 3.46, *p* = 0.03], RAVLT Delayed Recall [*F*(2, 2995) = 5.07, *p* = 0.006], Digit Span Forward Score [*F*(2, 1471) = 3.32, *p* = 0.04], Digit Span Forward Span [*F*(2, 1466) = 2.91, *p* < 0.05], and WMH volume [*F*(2, 2277) = 4.52, *p* = 0.01]. Both the ARB-ACEI group and the HTN-Other group performed worse than normotensive subjects on the RAVLT measures (Immediate Recall both *p =* 0.03, Delayed Recall both *p* < 0.01). The ARB-ACEI group also performed worse than normotensive subjects on Digit Span Forward Score and Digit Span Forward Span (both *p* = 0.02). They performed worse than the HTN-Other group on Digit Span Forward Score (*p* = 0.03). Both the ARB-ACEI and the HTN-Other groups demonstrated greater WMH volume than normotensive subjects (ARB-ACEI *p* = 0.005, HTN-Other *p* = 0.04).

#### BBB-crossing capability

Given that the capability of medications to cross the BBB is related to drug efficacy, a group using BBB-crossing ARBs (ARB crossers) was compared with a group using non-BBB-crossing (ARB noncrossers), a group using all other antihypertensive medications (HTN-Other), and normotensive subjects. Significant time × group interactions were found for three measures of memory: RAVLT Delayed Recall [*F*(3, 1759) = 5.07, *p* = 0.002], Logical Memory Immediate Recall [*F*(3, 1772) = 3.05, *p* = 0.03], and Logical Memory Delayed Recall [*F*(3, 1766) = 3.39, *p* = 0.02]. The HTN-Other group performed significantly worse over time than both the ARB crossers group and normotensive subjects on all three measures (all *p* < 0.05). On RAVLT Delayed Recall, the ARB noncrossers group performed significantly worse than the HTN-Other group (*p* = 0.005), the ARB crossers group (*p* = 0.0003), and normotensive subjects (*p* = 0.03).

**Fig. 4:**
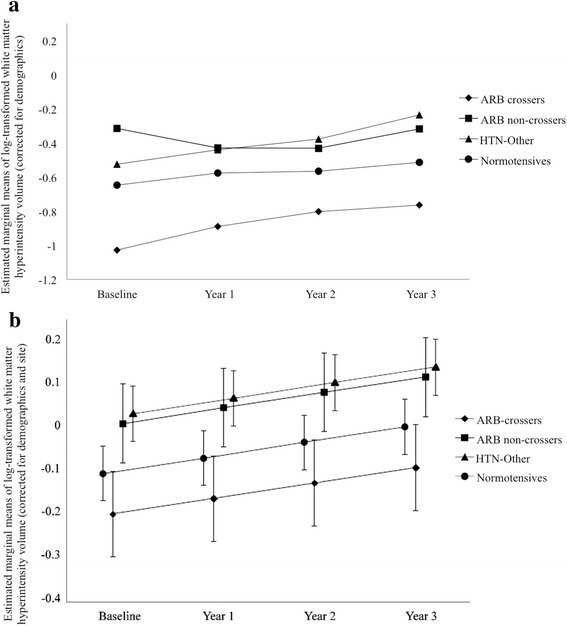
White matter hyperintensity (WMH) volume over the 3-year follow-up period. **a** estimated marginal means after correction for demographics only, **b** means adjusted for demographics and Alzheimer’s Disease Neuroimaging Initiative site. After all corrections, the participants who took other antihypertensive drugs that were not angiotensin II receptor blockers (HTN-Other) showed significantly greater WMH volume over time compared with users of blood-brain barrier (BBB)-crossing angiotensin II receptor blocker users (ARBs; BBB crossers) and normotensive subjects. Within the ARB users, the BBB crossers group had less WMH volume than users of non-BBB-crossing ARBs

There were significant effects of group for RAVLT Recognition [*F*(3, 2988) = 2.80, *p* = 0.04] and WMH volume [*F*(3, 2275) = 5.28, *p* = 0.001]. The HTN-Other group performed worse than the ARB noncrossers group (*p* = 0.02) and normotensive subjects (*p* = 0.03) on RAVLT Recognition. As shown on Fig. 4, the HTN-Other group also had significantly greater WMH volume than the ARB crossers group (*p* = 0.008) and normotensive subjects (*p* = 0.0006). Within the ARB user group, the ARB crossers group had less WMH volume than the ARB-noncrossers group (*p* = 0.05).

#### BBB-crossing RAAS medications

A group using BBB-crossing RAAS medication users (BBB crossers) was compared with a group using non-BBB-crossing RAAS medications (BBB noncrossers), a group of users of all other antihypertensive medications (HTN-Other), and normotensive subjects. Significant time × group interactions were found for two measures of memory—RAVLT Delayed Recall [*F*(3, 1752) = 4.58, *p* = 0.003] and Logical Memory Delayed Recall [*F*(3, 1759) = 2.66, *p* = 0.05]—as well as for WMH volume [*F*(3, 1135) = 2.60, *p* = 0.05]. Users of BBB-crossing RAAS medications performed better than all other groups, including normotensive subjects, on RAVLT Delayed Recall over time (BBB noncrossers and normotensive subjects *p* = 0.001, HTN-Other *p* = 0.01). Additionally, they were no different from normotensive subjects on Logical Memory Delayed Recall over time (*p* = 0.14), unlike the BBB noncrossers group, which performed significantly worse than normotensive subjects (*p* = 0.02). Users of other antihypertensive medications showed a trend toward performing worse than normotensives on this measure (*p* = 0.07). The BBB crossers group also had more WMH volume over time than normotensive subjects (*p* = 0.01) but less WMH volume than the BBB-noncrossers group (*p* = 0.03).

## Discussion

Findings of the present study provide support for our hypothesis that ARBs may confer protective effects on cognition greater than those of other antihypertensive drugs. At baseline, participants taking other antihypertensive medications exhibited worse memory and executive function than normotensive subjects, but ARB users did not differ from normotensive subjects on any measure of memory function and demonstrated better recognition memory than those taking other antihypertensive medications. Over a 3-year follow-up period, those taking other antihypertensive drugs performed significantly worse than normotensive individuals on immediate and delayed recall of a short story, but ARB users were no different from normotensive individuals on memory testing. These cognitive differences are underscored by the fact that both hypertensive groups exhibited elevated vascular risk factor burden relative to the normotensive group, as evidenced in higher systolic and diastolic blood pressure, pulse pressure, and BMI scores, as well as prior diagnoses of cardiovascular disease, dyslipidemia, and type 2 diabetes. Therefore, the hypertensive groups as a whole were potentially more vulnerable to cognitive impairment, but only the subset taking ARBs demonstrated expected practice effects on memory testing. The cognitive differences between the medicated hypertensive groups are especially notable, given that the ARB group had significantly more participants diagnosed with type 2 diabetes, which has been associated with a 1.5- to 2.5-fold greater risk of dementia [40]. Even with this added risk factor, the ARB users demonstrated preserved memory function over 3 years of follow-up.

Consistent with prior work suggesting that ACEIs may be less beneficial than ARBs for cognition, in the present study we found that when ARB users were combined with users of ACEIs, beneficial effects on memory were no longer observed. Li and colleagues [41], who examined 819,491 predominantly male participants over the age of 65 years with cardiovascular disease in a large epidemiological study, reported that ARB use was associated with a significant reduction in the incidence and progression of dementia and Alzheimer’s disease in comparison to ACEIs and other cardiovascular drugs. Additionally, researchers in a nested case-control study of adults aged 60 years or older, who had either Alzheimer’s disease, vascular dementia, or unspecified/other dementia, reported that patients taking ARBs and ACEIs had 53% and 24% lower risks of Alzheimer’s disease, respectively, than those taking other antihypertensive medications [42]. On one hand, whereas ACEIs reduce the amount of free angiotensin II and decrease damaging angiotensin II receptor type 1 (AT1) receptor activity, they also reduce beneficial angiotensin II receptor type 2 (AT2) receptor activity. On the other hand, given that ARBs block AT1 receptors and not the production of angiotensin II, they promote AT2 receptor activity and are “angiotensin-converting enzyme (ACE)-sparing,” in theory allowing ACE to continue its suggested Aβ-degrading function, unlike ACEIs [43].

Nevertheless, it must be noted that investigators in large clinical intervention trials such as ONTARGET and TRANSCEND have not found significant differences in the incidence of dementia between groups taking ARBs, ACEIs, or placebo [44]. Reasons for the discrepancies in these results and the results of the present study may include differences in sample characteristics. In both ONTARGET and TRANSCEND, researchers examined patients with high vascular disease burden, evidenced by established atherosclerotic cardiovascular disease or diabetes with end-organ damage. Such patients are likely to develop dementia with vascular etiology. In contrast, participants in the ADNI sample were excluded on the basis of criteria that limit cerebrovascular disease. Hence, the ratio of vascular to Alzheimer’s disease pathology was likely greater in the samples in ONTARGET and TRANSCEND. The lack of distinction between dementia subtypes in these studies may have obscured any beneficial effects that hypertensive treatment may have had on specific types of dementia, which is important, given the differential effects of ARBs and ACEIs on Aβ and Alzheimer’s disease pathology. Additionally, the mean ages of participants in ONTARGET and TRANSCEND were 66.4 and 66.9 years, respectively, which are 7–8 years younger than the mean age of the patients with hypertension in our sample and approximately a decade younger than the age at which rates of dementia increase [44]. Because Alzheimer’s disease risk increases with age, our sample likely had greater Alzheimer’s disease pathology, which may have contributed to our results of group differences in memory testing. The intervention trials also relied heavily on achieved scores and changed scores on a single measure (MMSE) to diagnose cognitive impairment and cognitive decline. Our study benefits from assessment of cognition through a comprehensive neuropsychological battery that may have been better suited to discerning changes across a range of domains.

Our study also found associations between the use of BBB-crossing ARBs and better performance across the 3-year follow-up on three measures of memory (RAVLT Delayed Recall, Logical Memory Immediate Recall, and Logical Memory Delayed Recall) in comparison to the HTN-Other group, which in turn performed worse than normotensive subjects. Interestingly, on the first measure, users of BBB-crossing ARBs performed better than users of non-BBB-crossing ARBs. Group differences were also found in WMH volume, with users of BBB-crossing ARBs showing significantly less WMH volume than all other treated subjects with hypertension. A combined group of users of BBB-crossing ARBs and BBB-crossing ACEIs demonstrated better performance on delayed list-learning recall over a 3-year follow-up period than users of non-BBB-crossing ARBs and ACEIs and had less WMH volume than this group. Therefore, among the various antihypertensive medications affecting the RAAS, those with the capacity to cross the BBB were associated with better memory over time and less WMH volume than their non-centrally acting counterparts. However, our findings were somewhat mixed with regard to memory because users of non-BBB-crossing ARBs showed better memory on some test scores, suggesting that among ARBs, both the BBB-crossing and non-BBB-crossing varieties may be of benefit.

Prior work has similarly examined differences in cognition among users of BBB-crossing and non-BBB-crossing ACEIs. Ohrui and colleagues [45], in examining differences among hypertensive patients taking BBB-crossing ACEIs (captopril or perindopril) and patients taking non-BBB-crossing ACEIs (imidapril or enalapril), calcium channel blockers, β-blockers, and diuretics, reported significantly lower risk of Alzheimer’s disease in the group taking BBB-crossing ACEIs than in those taking the non-BBB-crossing variety (odds ratio 0.25, 95% confidence interval 0.08–0.75, *p* = 0.014). Other studies have also found associations between BBB-crossing ACEIs and less severe cognitive decline in MMSE and modified MMSE scores when compared with non-BBB-crossing ACEI use [45, 46]. Nevertheless, these results need confirmation in an RCT of BBB-crossing hypertensive drugs in dementia prevention or reduction.

The protective mechanism responsible for preserved memory among ARB users remains uncertain and is likely to involve a complex interaction of multiple pathways. In the present study, only BBB-crossing ARB users showed significantly lower WMH volume relative to other antihypertensive medication users, who exhibited greater white matter lesion burden than normotensive subjects. Notably, when ARB users were combined with users of ACEIs, beneficial effects on memory and WMH volume disappeared: The combined ARB-ACEI group, as well as users of all other antihypertensive drugs, performed worse on measures of immediate and delayed recall and had greater WMH volume than normotensive subjects. The ARB-ACEI group also performed worse than normotensive subjects on measures of attention. Therefore, compared with other drugs impacting the RAAS, ARBs appear to be associated with maintenance of memory and attention, as well as amount of white matter lesions, which can disrupt frontal-subcortical connections critical to memory retrieval functions [47].

ARBs may also exert their effects through improving cerebral blood flow and reducing ischemia through their influence on the RAAS. The RAAS regulates blood pressure through its effects on fluid homeostasis and vascular tone. Although the RAAS is more commonly associated with endocrine and vascular-renal functions, research has also established the presence of a paracrine RAAS within the central nervous system that acts largely independently of peripheral function [48]. The RAAS in the brain is believed to be involved in processes beyond mere blood pressure control, including processes of learning and memory [49]. ARBs target the locally acting brain RAAS and block AT1 receptors, allowing greater AT2 receptor binding [32], thus exerting their effects by both interrupting AT1 receptor activity and promoting AT2 receptor activity. AT1 receptor activity includes the generation of free radicals and the activation of multiple inflammatory pathways, all of which lead to tissue damage [50]. Greater AT2 receptor activity decreases vasoconstriction, thus increasing cerebral blood flow, which is protective against inflammation and ischemia [51].

In a recent longitudinal analysis of cerebrospinal fluid biomarker data from ADNI, we reported an attenuation of cerebral amyloid retention and progression to dementia among older adults taking ARBs [52]; however, there were no cross-sectional differences in amyloid retention. The present study extends these findings by demonstrating maintenance of memory function and amount of WMH among ARBs users at baseline and 3-year follow-up. Together, these results present a complex picture in which ARBs may be protective of memory function by possibly stymieing effects of cerebral small vessel disease and/or Alzheimer’s disease pathology or otherwise improving neuronal function through direct actions on cerebral blood flow or neuronal RAAS receptors. The strength of these benefits may vary, depending on the use of other drugs impacting RAAS, whether the ARBs cross the BBB, and other individual differences such as age and vascular risk factor burden.

To our knowledge, this is the first study to examine performance on a comprehensive neuropsychological battery among users of various antihypertensive drugs, as well as the first study to investigate differences in cognitive trajectories among users of BBB-crossing and non-BBB-crossing ARBs. Our study benefits from the examination of performance on multiple tests measuring multiple cognitive domains. Other strengths of the present study are its longitudinal design, large sample size, and inclusion of neuroimaging markers of brain atrophy and WMH volume.

Despite these strengths, the present study is not without limitations. We did not account for multiple drug combinations, owing to sample size limits. We focused on the use of ARBs and were unable to fully disentangle the potential effects of ACEIs, independent of ARBs, owing to sample size limitations. Future studies are needed to determine the independent effects of ACEIs. Given the exploratory nature of this study, we did not account for multiple comparisons. Comparison of the baseline characteristics of participants who presented vs. those who did not present for the 3-year follow-up revealed that those who presented had significantly more MCI diagnoses, higher systolic and diastolic blood pressure, and higher rates of carotid artery disease (see Additional file 4). Therefore, participants who presented at follow-up may have had more concerns regarding their cognitive functioning and vascular health and may have biased results against a null effect. Owing to inconsistent recording of medication and medical history following baseline visits, it was impossible to determine whether participants switched between groups over time. Additionally, the ADNI sample is comprised of participants from over 50 sites in the United States and Canada with varied sampling biases. Thus, we accounted for local prescribing practice by clustering participants by site in our analyses. Participants were excluded on the basis of criteria that restricted cerebrovascular disease, limiting generalizability. Effects of white matter disease may be greater in the broader population. Further, we cannot rule out possible confounding by indication, because ARB use in itself may be a risk indicator for the severity of hypertension. ARBs are prescribed as supplementary drugs in older patients with hypertension that remains uncontrolled after use of other drugs [53]. However, if there had been confounding by indication, we would predict that the ARB users would demonstrate worse cognitive performance than the group taking other antihypertensive drugs, given that the ARB users had potentially more severe hypertension as well as a higher proportion of participants with type 2 diabetes. In light of this, the superior cognitive performance of the ARB group in comparison to users of other antihypertensive drugs, including those also impacting the RAAS, is therefore notable.

## Conclusions

Hypertensive participants demonstrated worse baseline memory and executive function and greater memory decline over 3 years follow-up than normotensive subjects, unless they were ARB users, who showed preserved memory compared with those taking other antihypertensive drugs. Users of BBB-crossing ARBs showed superior memory performance over time compared with other antihypertensive drug users and had less WMH volume. Users of BBB-crossing medications (ARBs or ACEIs) showed better list-learning memory performance over time than all other groups, including normotensive subjects, and had less WMH volume over time than users of non-BBB-crossing medications. These findings demonstrate that ARBs, especially those of the BBB-crossing variety, are associated with greater memory preservation and less WMH volume than other antihypertensive medications.

## Additional files

Additional file 1: Baseline medications. (DOC 29 kb)

Additional file 2: Baseline number of hypertensive medications. (DOC 29 kb)

Additional file 3: Flowchart of inclusion/exclusion of participants. (PNG 272 kb)

Additional file 4: Baseline characteristics of participants with missing vs. nonmissing data at 3-year follow-up visit. (DOC 92 kb)

## Abbreviations

ACE: Angiotensin-converting enzyme
ACEI: Angiotensin-converting enzyme inhibitors
ADNI: Alzheimer’s Disease Neuroimaging Initiative
ANOVA: Analysis of variance
APOE: Apolipoprotein E
ARB: Angiotensin II receptor blocker
AT1: Angiotensin II type 1
AT2: Angiotensin II type 2
Aβ: Amyloid-β
BBB: Blood-brain barrier
BMI: Body mass index
BNT: Boston Naming Test
BP: Blood pressure
HTN-ARB: Participants who took angiotensin II receptor blockers
HTN-Other: Participants who took other antihypertensive drugs that were not angiotensin II receptor blockers
HYVET-COG: Hypertension in the Very Elderly Trial assessing cognitive decline and dementia incidence
LM: Logical Memory
MCI: Mild cognitive impairment
MMSE: Mini Mental State Examination
MRC-Elderly: Medical Research Council Elderly study
MRI: Magnetic resonance imaging
ONTARGET: Ongoing Telmisartan Alone and in Combination with Ramipril Global Endpoint Trial
PROGRESS: Perindopril Protection against Recurrent Stroke Study
RAAS: Renin-angiotensin-aldosterone system
RAVLT: Rey Auditory Verbal Learning Test
RCT: Randomized controlled trial
SHEP: Systolic Hypertension in the Elderly Program
SYST-EUR: Systolic Hypertension in Europe
TIA: Transient ischemic attack
TRANSCEND: Telmisartan Randomised AssessmeNt Study in ACE iNtolerant subjects with cardiovascular Disease
WAIS-R: Wechsler Adult Intelligence Scale–Revised
WMH: White matter hyperintensities
WMS-R: Wechsler Memory Scale–Revised
