# Supplementary material for: Memory is preserved in older adults taking AT1 receptor blockers
Source: Alzheimers Res Ther. 2017 Apr 26;9:33. doi: 10.1186/s13195-017-0255-9 (PMC5405458; doi:10.1186/s13195-017-0255-9)
Supplement: Supplementary file 1 — Baseline medications. (DOC 29 kb) [file 13195_2017_255_MOESM1_ESM.doc]

**Additional File 1: Baseline Medications**

| **Medications (used in addition to ARBs for the HTN-ARBs group)** | **HTN-ARBs (n=183)** | **HTN-Other (n=621)** |
| --- | --- | --- |
| ACE-inhibitors | 9 (4.9%) | 291 (46.9%) |
| Beta-blockers | 41 (22.4%) | 239 (38.5%) |
| Calcium channel blockers | 45 (24.6%) | 168 (27.1%) |
| Alpha-2-agonists | 3 (1.6%) | 2 (0.3%) |
| Diuretics | 77 (42.1%) | 252 (40.6%) |
| Direct vasodilators | 10 (5.5%) | 38 (6.1%) |
