# Supplementary material for: Memory is preserved in older adults taking AT1 receptor blockers
Source: Alzheimers Res Ther. 2017 Apr 26;9:33. doi: 10.1186/s13195-017-0255-9 (PMC5405458; doi:10.1186/s13195-017-0255-9)
Supplement: Supplementary file 2 — Baseline number of hypertensive medications. (DOC 29 kb) [file 13195_2017_255_MOESM2_ESM.doc]

**Additional File 2: Baseline Number of Hypertensive Medications**

| **Number of hypertensive medications** | **HTN-ARBs (n=183)** | **HTN-Other (n=621)** |
| --- | --- | --- |
| 1 | 59 (32.2%) | 350 (56.4%) |
| 2 | 78 (42.6%) | 188 (30.3%) |
| 3 | 35 (19.1%) | 70 (11.3%) |
| 4 | 8 (4.4%) | 11 (1.8%) |
| 5 | 2 (1.1%) | 2 (0.3%) |
| 6 | 1 (0.5%) | 0 (0%) |
