# Supplementary figures and images for: Memory is preserved in older adults taking AT1 receptor blockers
Source: Alzheimers Res Ther. 2017 Apr 26;9:33. doi: 10.1186/s13195-017-0255-9 (PMC5405458; doi:10.1186/s13195-017-0255-9)

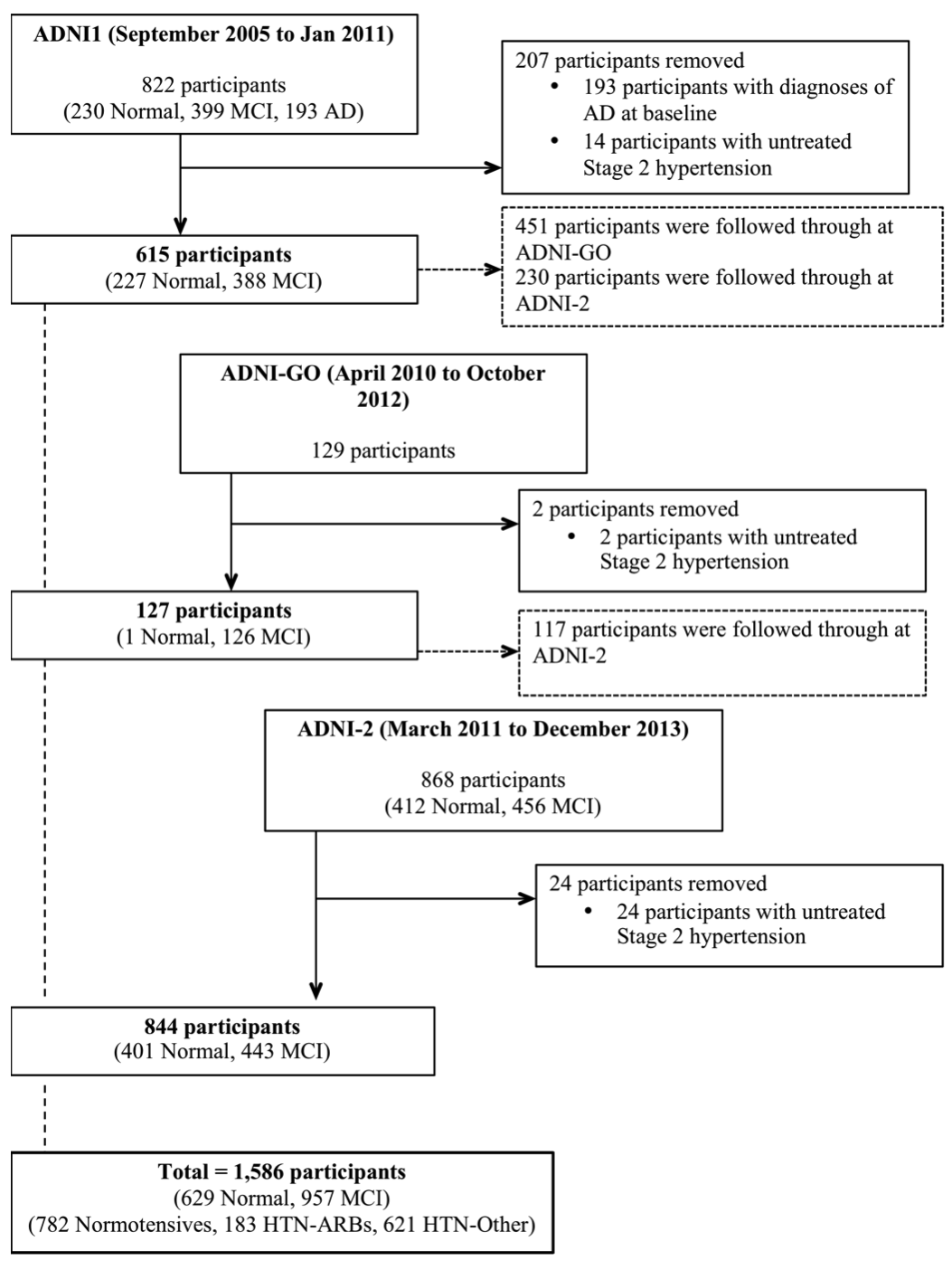

Supplement: Supplementary file 3 — Flowchart of inclusion/exclusion of participants. (PNG 272 kb) [file 13195_2017_255_MOESM3_ESM.png]
