# Supplementary material for: Memory is preserved in older adults taking AT1 receptor blockers
Source: Alzheimers Res Ther. 2017 Apr 26;9:33. doi: 10.1186/s13195-017-0255-9 (PMC5405458; doi:10.1186/s13195-017-0255-9)
Supplement: Supplementary file 4 — Baseline characteristics of participants with missing vs. nonmissing data at 3-year follow-up visit. (DOC 92 kb) [file 13195_2017_255_MOESM4_ESM.doc]

**Additional File 4: Baseline Characteristics of Participants with Missing vs. Non-missing Data at 3-year Follow-up Visit**

| **Clinical/**  **Demographic** | Missing at 3-year follow-up visit  n = 822 | Presented at 3-year follow-up visit  n = 764 | F or χ ^2^ | p-value |
| --- | --- | --- | --- | --- |
| Age, yrs | 73.9 (6.7) | 73.3 (7.1) | 2.161 | 0.142 |
| Education, yrs | 16.1 (2.9) | 16.1 (2.7) | 0.026 | 0.873 |
| Sex (% men) | 53.7% | 56.8% | 1.260 | 0.281 |
| APOE Genotype  (% ε4+) | 43.0% | 42.3% | 0.061 | 0.806 |
| Diagnosis (% MCI) | 47.5% | 72.1% | 80.985 | **< .001** |
| BMI (kg/m^2^) | 27.0 (4.9) | 27.1 (4.5) | 0.062 | 0.804 |
| Systolic BP (mmHg) | 135.1 (15.6) | 132.8 (15.9) | 6.449 | **0.011** |
| Diastolic BP (mmHg) | 75.0 (9.2) | 73.6 (9.6) | 6.876 | **0.009** |
| Pulse pressure (mmHg) | 60.1 (14.4) | 59.2 (14.3) | 1.141 | 0.286 |
| **Vascular risk factors** |  |  |  |  |
| Cardiovascular disease | 12.9% | 11.3% | 0.851 | 0.356 |
| Dyslipidemia | 50.8% | 45.9% | 3.023 | 0.082 |
| Type 2 diabetes | 8.4% | 8.1% | 0.045 | 0.833 |
| Atrial fibrillation | 3.4% | 3.3% | 0.011 | 0.917 |
| Carotid artery disease | 0.0% | 1.3% | 7.031 | **0.008** |
| TIA / minor stroke | 2.8% | 2.9% | 0.005 | 0.945 |

Data are summarized as Mean (Standard Deviation), unless otherwise indicated. Significant differences (*p* < .05) among medication groups are indicated in bold.
